# Highly efficient glycolysis of polyethylene terephthalate catalyzed by a Zn-modified 5A zeolite prepared through ion exchange

**DOI:** 10.1039/d6ra08028f

**Published:** 2026-11-04

**Authors:** Haibo Wang, Xiuqi Wang, Zixin Lin, Haoyu Yao

**Affiliations:** a Key Laboratory of Marine Chemistry Theory and Technology (Ministry of Education), College of Chemistry and Chemical Engineering, Ocean University of China Qingdao China linzixin@ouc.edu.cn; b Qingdao Institute of Bioenergy and Bioprocess Technology, Chinese Academy of Sciences Qingdao China yaohy@qibebt.ac.cn; c Shandong Energy Institute Qingdao China

## Abstract

Polyethylene terephthalate (PET) is one of the most widely produced polyesters worldwide, but its low recycling efficiency has caused serious environmental pollution and resource waste. Glycolysis is considered an optimal method for closed-loop PET recycling because it works under mild conditions and allows direct recovery of the valuable monomer bis(2-hydroxyethyl) terephthalate (BHET). Catalysts are very important in glycolysis, but most zinc-based solid catalysts reported so far have high costs and are not stable enough, and this makes them hard to use in industrial applications. To solve these problems, this study prepared a series of zinc-modified zeolite catalysts (ZnX-LTA/*N*&*T*) using industrial 5A zeolite (LTA) as the support with a simple ion exchange method, and evaluated the effects of the zinc precursor types, ion-exchange cycles, and calcination temperatures on the structure and performance of the catalysts. ZnCl_2_-LTA/3&400 (prepared with using ZnCl_2_ as precursor, calcination at 400 °C, and three ion exchange cycles) showed the best catalytic performance with 87.33% PET conversion and 74.23% BHET yield, and also maintained well stability after few recycling runs. Kinetic studies showed that the PET glycolysis followed shrinking core model with activation energy of 184.41 kJ mol^−1^. In addition, this catalyst also exhibited excellent activity for depolymerizing various real post-consumer PET wastes (films, trays, and beverage bottles), achieving 100% conversion for PET films with a BHET yield of up to 81.93%.

## Abstract 2

Polyethylene terephthalate (PET) is one of the most widely produced polyesters worldwide, but its low recycling efficiency has caused serious environmental pollution and resource waste.

## Graphical abstract

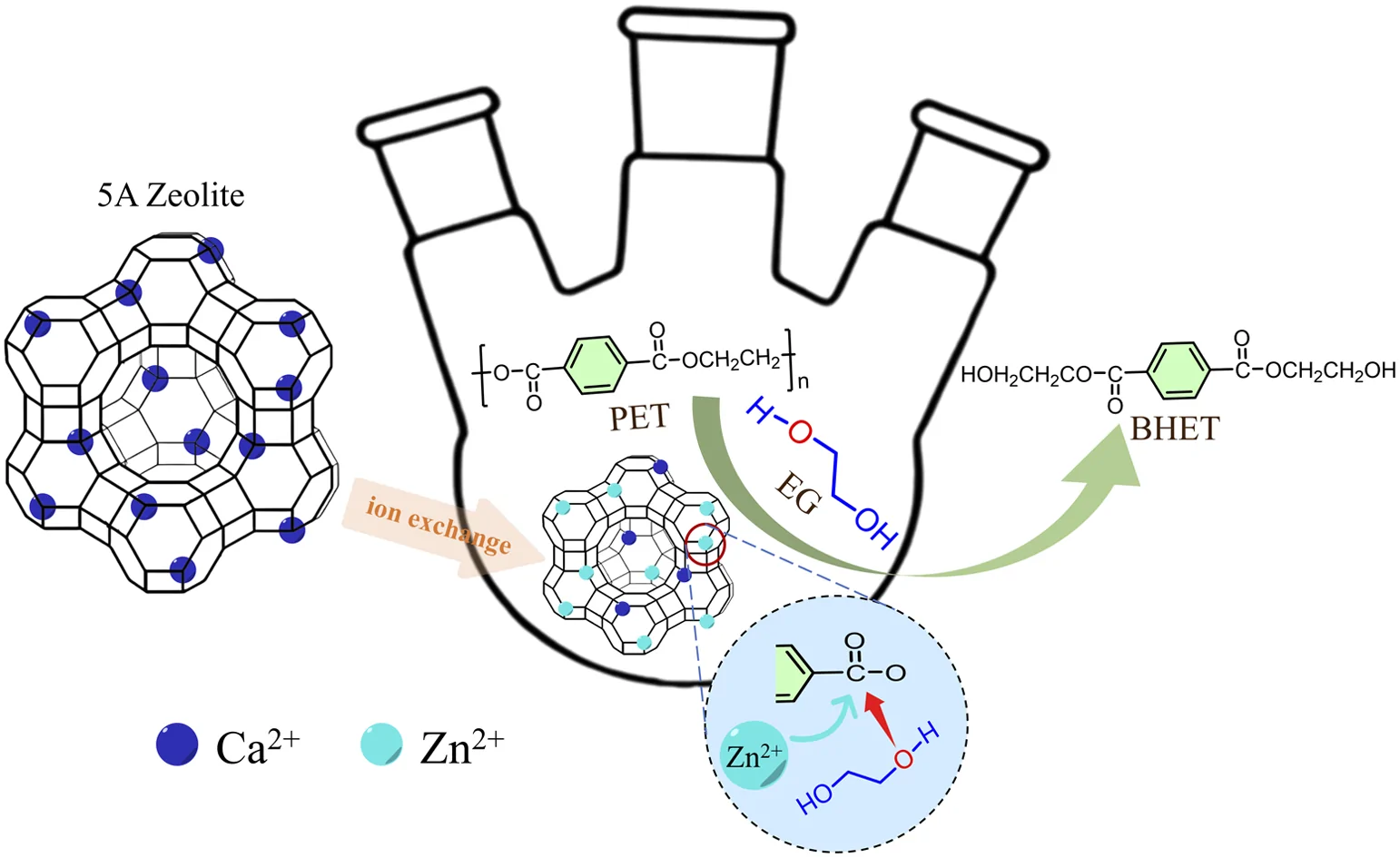

## 1. Introduction

Polyethylene terephthalate (PET) is one of the most widely used synthetic polymers worldwide, with yearly production exceeding tens of millions of tons.^1^ Thanks to excellent mechanical properties, chemical stability and low cost, it has been widely applied in fields such as packaging materials,^2^ textile fibers,^3^ construction components,^4^ and medical equipment.^5^ Despite progress in recycling technologies, we still cannot recover waste PET adequately.^6^ This inadequate recovery causes pollution because landfill disposal or ecological release, and makes white pollution worse. Current PET recycling technologies are mainly divided in mechanical and chemical.^7,8^ Mechanical recycling encounters problems such as polymer chain breakage and contamination accumulation, these limit PET recovery. Chemical recycling^9^ include glycolysis,^10–12^ methanolysis,^13,14^ hydrolysis,^15,16^ and ammonolysis,^17,18^ enable PET depolymerization into pure monomers or intermediates, facilitating closed-loop cycles. Among these technologies, glycolysis is especially potential for industrial promise because its modest reaction conditions, simple operation and direct generation of the bis(2-hydroxyethyl) terephthalate (BHET) which is a precursor used for the subsequent polymerization.^19^

The choice of the catalytic systems has a critical effect in the glycolysis of PET in terms of reaction efficiency, product selectivity and also the process economics. Presently, catalytic systems^20^ mainly contain homogeneous metal salts, ionic liquids, and deep eutectic solvents (DESs). Chiao *et al.*^21^ used zinc plates from waste batteries as precursors, active species such as zinc ethanoate and zinc oxide are generated *in situ* in ethylene glycol (EG). This method got 89.7% PET conversion and 77.7% BHET yield, which were on the same performance level as the conventional zinc acetate catalysts. But the high solubility of generated zinc species in EG are very difficult for catalyst separation and recovery. Similarly, Chen *et al.*^22^ made a mixed ionic liquid system with [Hmim]ZnCl_3_ and [Hmim]CoCl_3_, and attained complete PET conversion and 87.1% BHET yield at mild conditions (190 °C). But this good performance has problems such as complex synthesis protocols, high costs, and recovering through high-energy-consuming methods like vacuum distillation. Another way is from Lu *et al.*,^23^ they constructed a series of l-proline (Pro)-based deep eutectic solvents for PET glycolysis with 1,4-butanediol (BDO). Under best conditions, PET broke down completely with 67.1% BHET yield. Although DES offer the advantages of low-cost raw materials and straightforward preparation procedures, the industrial application is still limited because of the requirement of high catalyst loading and the relatively low product selectivity. These bottlenecks such as the difficulty in recycling, high cost and low selectivity make these catalytic systems unable to meet industrial-scale demands. In contrast, heterogeneous catalysts are regarded as a key direction to break through the engineering bottleneck of PET chemical recycling due to their inherent advantages such as efficient solid–liquid separation, excellent cycling stability and environmental friendliness. Thus, developing new heterogeneous catalysts with the facile recyclability, low cost and high catalytic activity, has become the research focus in the field of PET recycling.

To establish a rational design for efficient PET glycolysis catalysts, a critical comparison of existing heterogeneous catalyst systems is essential. As summarized in Table 1, the catalytic performance is governed by both the intrinsic activity of the metal species and the structural properties of the support. First, regarding the active metal center, control experiments on identical SBA-15 supports unequivocally demonstrate the superiority of zinc. Under the same loading (5 wt%), ZnO/SBA-15 achieves a remarkable BHET yield of 91.55% with a TOF of 155.08 h^−1^, significantly outperforming Co (48.5%), Mg (71.8%), Ni (6.51%), and Cu (0.89%) based counterparts. This confirms that Zn^2+^ Lewis acid sites possess the optimal electronic structure for activating ester carbonyls. Second, the choice of support is equally critical in determining the catalyst's potential. A comparison of different porous materials reveals that molecular sieves generally exhibit superior catalytic efficiency compared to other supports like MOFs. For instance, Zn-based molecular sieves (ZnO/SBA-15 and Zn/MCM-41) achieve yields exceeding 77% and TOFs up to 185.78 h^−1^, outperforming or matching the activity of ZnCl_2_/ZIF-8 (MOF) while offering better structural robustness. As a typical molecular sieve, the 5A zeolite (LTA type) has significant potential as an ideal catalyst support due to its well-defined crystalline architecture, excellent thermal resilience, template-free synthesis, and established industry infrastructure. To tackle the problem of active component leaching, our team adopted the ion exchange strategy based on the previous successful experience in constructing highly stable Cs/LTA catalysts.^29^ This method exploits the unique Ca^2+^ site in the 5A zeolite framework for the selective substitution and secure strong anchoring of the Zn^2+^ ions, to establish a durable metal–support synergistic structure which can prevent the migration and leaching. Importantly, beyond the catalytic performance, the economic feasibility of this approach is noteworthy. As detailed in the preliminary cost analysis (Table S1), the estimated preparation cost of the ZnCl_2_-LTA/3&400 catalyst is approximately $4.13 per kg. This low cost is primarily attributed to the use of commercially available LTA (5A) zeolite as a support and a simple ion-exchange synthesis route that avoids expensive templating agents or organic linkers. In contrast, template-assisted mesoporous silica (SBA-15, MCM-41) and MOF-based (ZIF-8) catalysts typically incur significantly higher material and synthesis costs (Table S2). Specifically, the estimated cost of our catalyst is roughly 4–15 times lower than that of reported Zn-based heterogeneous alternatives. Besides, current study in this field face two critical limitations, the preparation of the catalyst had only depended on single variable optimization without global parameter tuning; and the evaluation system is only confined to ideal pure PET without test of the performance adaptability to the complex real waste PET.

**Table 1:** Comparison of catalytic performance for PET glycolysis over different heterogeneous systems

Catalyst system	Support type	Metal	Loading (wt%)	Temp. (°C)	Time (h)	Yield (%)	TOF (h^−1^)	Ref.
ZnO/MCM-41	Molecular sieve	ZnO	7	190	2.0	77.88	47.12	24
Zn/MCM-41-25	Molecular sieve	Zn	8	200	1.8	80.09	185.78	24
ZnO/SBA-15	Molecular sieve	ZnO	5	197	1.0	91.55	155.08	25
CoO/SBA-15	Molecular sieve	CoO	5	197	0.5	48.50	151.29	25
NiO/SBA-15	Molecular sieve	NiO	5	197	0.5	6.51	20.24	25
CuO/SBA-15	Molecular sieve	CuO	5	197	0.5	0.89	2.95	25
MgO/SBA-15	Molecular sieve	MgO	5	197	0.5	71.80	120.46	25
MgO/Y_2_O_3_	Metal oxide	MgO	10	185	2.3	84.65	78.90	26
MgO/SiO_2_	SiO_2_	MgO	10	190	2.0	95.67	100.31	27
ZnCl_2_/ZIF-8	MOF	ZnCl_2_	—	195	0.4	83.20	—	28

Building upon this foundation, this study systematically synthesized a series of Zn-modified 5A zeolite catalysts by ion exchange method. Through varying the types of zinc precursor, ion exchange cycles, and calcination temperatures, we thoroughly investigate the structure–performance correlations and to find out the best catalytic configuration. After that, we further optimize the reaction conditions and combine the shrinking core model to analyze the reaction kinetics mechanism. Finally, we verified universality and practical applicability of this catalyst by the various types of waste PET. In conclusion, this work presents a heterogeneous catalytic solution with high performance and industrial feasibility for PET glycolysis, aiming to promote the development of PET chemical recycling technology towards large-scale and sustainable applications.

## 2. Experimental

### 2.1. Materials

Commercial PET pellets were obtained from DuPont Company (USA), mechanically comminuted, and sieved to a particle size range of 20–60 mesh. Post-consumer PET waste materials, comprising pallets, films, and transparent beverage bottles, were subjected to a standardized pretreatment involving washing, drying, shredding, and subsequent sieving to achieve the same 20–60 mesh particle size specification. The following analytical-grade chemical reagents were procured: zeolite 5A (80–100 mesh), anhydrous zinc acetate [Zn(CH_3_COO)_2_], anhydrous zinc sulfate (ZnSO_4_), and zinc chloride (ZnCl_2_) from Shanghai Macklin Biochemical Technology Co., Ltd; while zinc nitrate hexahydrate [Zn(NO_3_)_2_·6H_2_O] and EG were supplied by Sinopharm Chemical Reagent Co., Ltd.

### 2.2. Catalyst preparation

The ZnX-LTA/*N*&*T* zeolite catalyst were synthesized using an ion exchange methodology. Initially, a 5A zeolite precursor was subjected to ion exchange with various zinc salts (X = Cl^−^, NO_3_^−^, CH_3_COO^−^, SO_4_^2−^) under ultrasonic assistance at 70 °C for 6 h. The resulting suspension was vacuum-filtered, rinsed thoroughly with deionized water, and subsequently dried in an oven at 100 °C for 5 h. Thermal activation was carried out in a muffle furnace at temperatures ranging from 200 to 800 °C (200, 400, 500, 600, and 800 °C) for 6 h with a controlled heating rate of 10 °C min^−1^. This protocol was systematically repeated across multiple ion exchange cycles (*N* = 1, 2, 3, 4, 5).

### 2.3. Catalyst characterization

The X-ray diffraction (XRD) patterns were recorded on a Rigaku SmartLab diffractometer using Cu Kα radiation (*λ* = 0.15406 nm) over a 2*θ* range of 5–80° at a scan speed of 20° min^−1^. Nitrogen adsorption–desorption isotherms were measured at −196 °C using a Micromeritics ASAP 2460 analyzer. Prior to analysis, samples were degassed under high vacuum (<2.66 Pa) at 150 °C for 6 h. The specific surface area (*S*_BET_) was calculated using the BET method, and the pore size distribution was derived from the desorption branch using the BJH model. Fourier transform infrared (FT-IR) spectroscopy was performed on a Bruker Vertex 70v spectrometer within the wavenumber range of 400–4000 cm^−1^. Thermogravimetric analysis (TGA) was carried out using a Netzsch STA449F5 Jupiter thermal analyzer in a flow nitrogen atmosphere with a heating program from 30 to 800 °C at 10 °C min^−1^. The morphological features of the catalysts were examined by field emission scanning electron microscopy (SEM) using an Hitachi S-4800 cold cathode instrument.

### 2.4. Glycolysis of PET

A mixture of PET, EG and catalyst was charged at specified mass ratio in a three-neck round-bottom flask with stirring rotor. A mercury thermometer was put in the left neck and the right neck was sealed with a glass stopper. The flask was put in the temperature-controlled oil bath. After 6 h of heating, hot filtration was made with deionized water at 90 °C, to make sure eliminating catalyst and residues completely from the flask. The isolated solid residue was dried in oven at 90 °C and the filtrate was afterwards secondary filtered following thermal treatment in 90 °C water bath (30 min). Refrigerating the filtrate at −4 °C for 12 hours. After that, the BHET crystals that formed were collected by filtration and dried at 70 °C. For the quantitative of the glycolysis performance in this study the PET conversion efficiency and the BHET yield were quantified according to the following equations:1
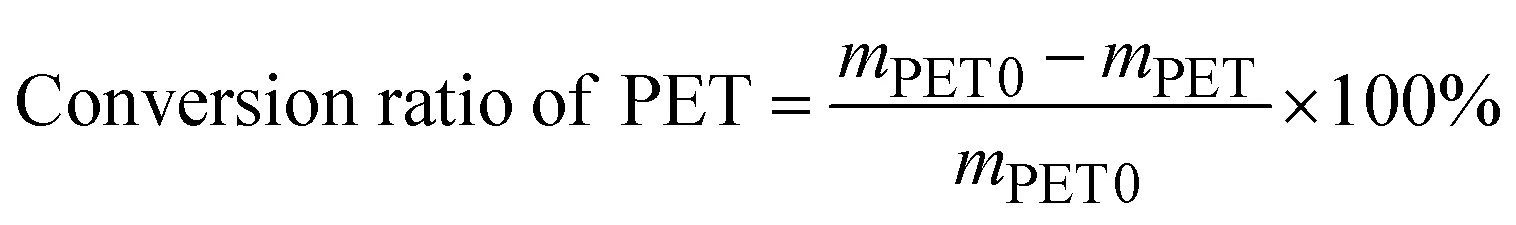
2
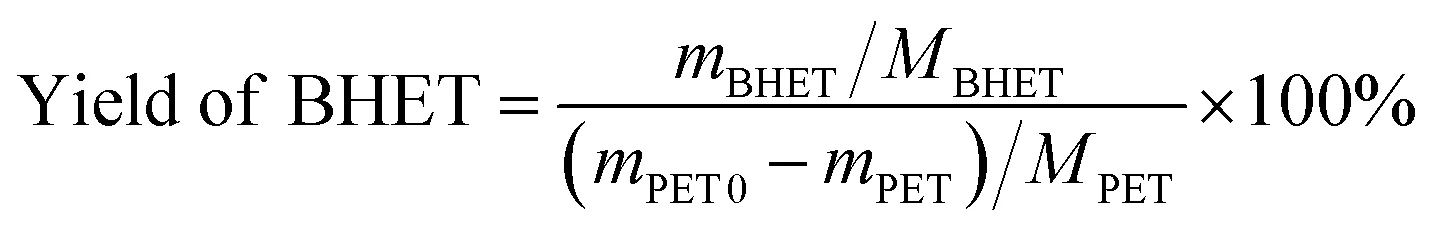
where *m*_PET0_, *m*_PET_ are the mass of PET before the reaction and after the reaction, respectively; *m*_BHET_ is the mass of BHET measured in the experiments; with *M*_BHET_ (254 g mol^−1^), *M*_PET_ (192 g mol^−1^) the molar mass of BHET and PET.

All catalytic glycolysis experiments were performed in duplicate, and the reported conversions and yields represent average values.

## 3. Results and discussion

### 3.1. Characterization of ZnX-LTA/*N*&*T*

The main difference between ZnX-LTA/*N*&*T* and the 5A zeolite is the introduction of Zn^2+^ into the LTA framework through ion exchange with varying numbers of exchange cycles. In Fig. 1a, the diffraction peaks of the pristine 5A zeolite at 2*θ* = 7.2°, 10.2° and 21.6°, which correspond to LTA structure (PDF #39-0222). With the increase of the ion-exchange cycles, the intensity of LTA diffraction peaks gradually weakens and almost disappear after the third exchange cycle, which means Zn^2+^ is highly loaded and significantly covers the original crystal structure. However, the characteristic LTA diffraction peaks remain discernible in all modified samples, indicating that the zeolite framework is largely preserved. The observed peak intensity reduction may arise from amorphous or highly dispersed Zn species on the zeolite surface rather than isomorphous substitution of framework atoms, as similarly reported for ion-exchanged Cs/LTA zeolites.^29^Fig. 1b shows that, under identical exchange conditions, the LTA diffraction peak intensity of the catalysts synthesized with ZnCl_2_ as zinc precursor have much lower than those derived from the Zn(NO_3_)_2_, Zn(CH_3_COO)_2_ and ZnSO_4_ systems, which confirm that the ZnCl_2_ system introduce more efficiently Zn^2+^ in the zeolite structure. Furthermore, the calcination temperature has a significant impact on the crystal phase structure (Fig. 1c): below 500 °C, only the LTA diffraction peak weakens; while at 800 °C, the LTA diffraction peaks completely disappear and new impurity peaks attributed to ZnAl_2_O_4_ appear at 2*θ* = 31.4° and 36.9°. It means that high calcination temperature causes irreversible collapse of the zeolite framework.

**Fig. 1:**
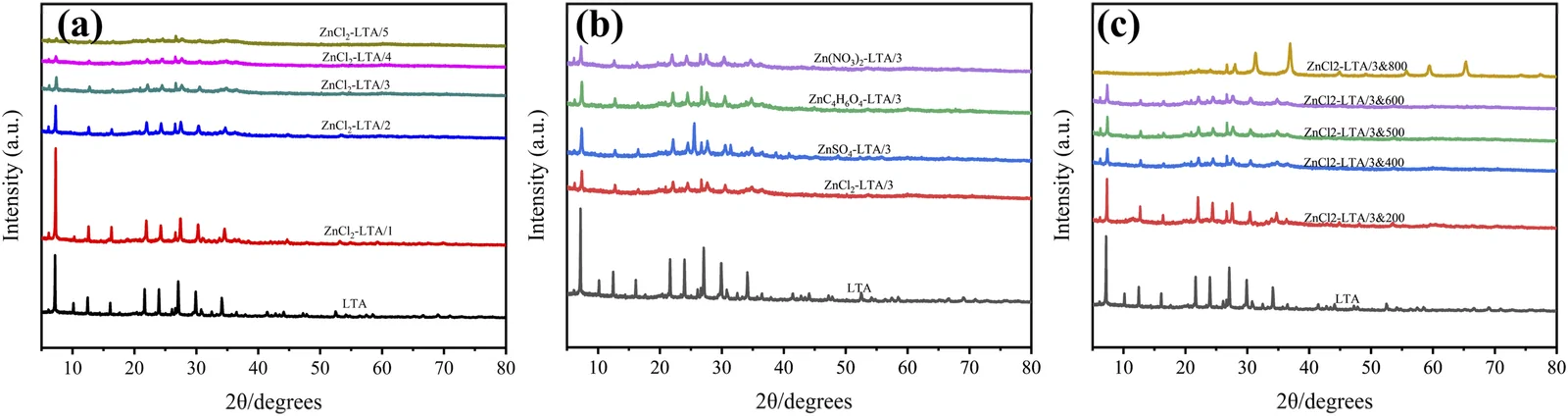
XRD curves of LTA with (a) number of ion exchanges; (b) zinc of different sources; (c) calcination temperature.

Fig. 2 shows the Fourier-transform infrared (FTIR) spectra of ZnX-LTA/*N*&*T*. All samples show the characteristic absorption peaks that correspond to the vibrational modes of the Si–O–Al framework in the 1400–800 cm^−1^. It is seen that the intensity of this peak significantly decreased after the introduction of Zn^2+^, and the samples prepared with ZnCl_2_ as zinc precursor showed the most pronounced attenuation of this peak. It means that the Zn^2+^ were successfully embedded into the zeolite framework or formed strong coordination bonds with the framework oxygen, and altering the vibration mode of the lattice. Fig. 2c also showed an increase in the intensity of the Si–O–Al peak. This observation is consistent with the formation of ZnAl_2_O_4_ spinel, as the consumption of framework Al during spinel formation can alter the local coordination environment and affect the Si–O–Al asymmetric stretching vibration.^30,31^

**Fig. 2:**
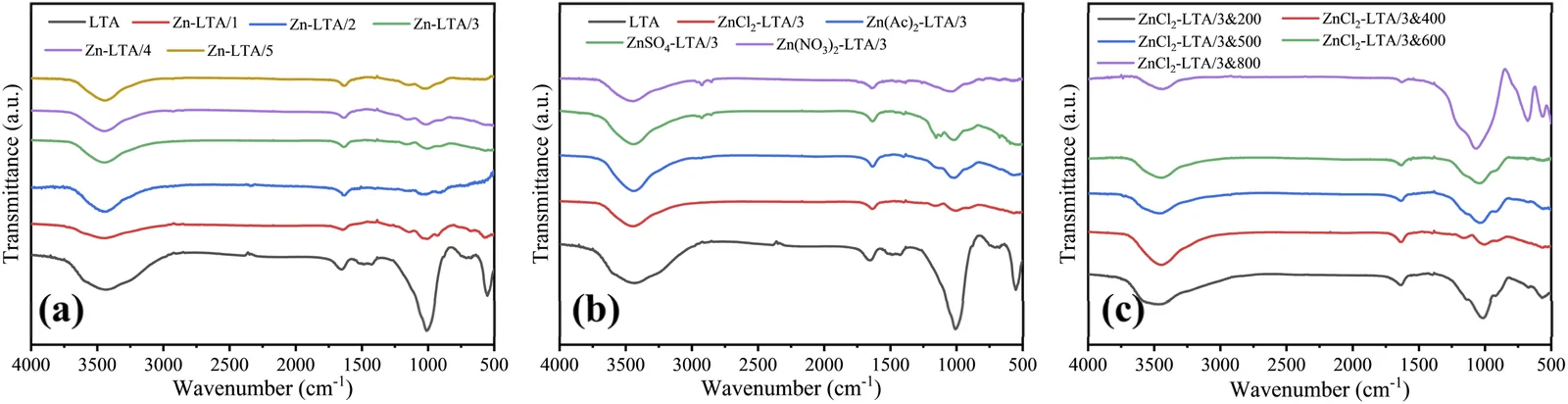
FTIR curves of LTA with (a) number of ion exchanges; (b) zinc of different sources; (c) calcination temperature.

The morphology and pore structure of the catalysts calcined at different temperature are shown in Fig. S1 and 3. The pristine 5A zeolite showed the uniform cubic particles with an average size about 2–3 µm. Its N_2_ adsorption isotherm displayed a characteristic type I isotherm, which proved its microporous structure. After Zn^2+^ modification and calcination, the particle morphology has undergone large changes. At calcination temperature of 200 °C and 400 °C, although the particles slightly aggregated but the overall dispersion was still good. However, when the calcination temperature rises to 500 °C or higher, serious sintering occurs, the particle size significantly increases and the surface becomes rough. All of Zn-modified samples showed the type IV isotherm (Fig. 3a), which proved that 5A zeolite have undergone a transformation from microporous to mesoporous structure. Especially the catalyst ZnCl_2_-LTA/3&400 has the most optimal pore structure with a specific surface area of 204.59 m^2^ g^−1^ and an average pore diameter of 3.22 nm (Table 2). As the calcination temperature raised from 400 °C to 800 °C, the specific surface area decreased sharply to 1.02 m^2^ g^−1^ and the average pore diameter increased abnormally to 21.71 nm, which was attributed to the complete collapse of the zeolite's framework and the serious particle sintering under high calcination temperature, as clearly shown in the SEM images (Fig. S1).

**Fig. 3:**
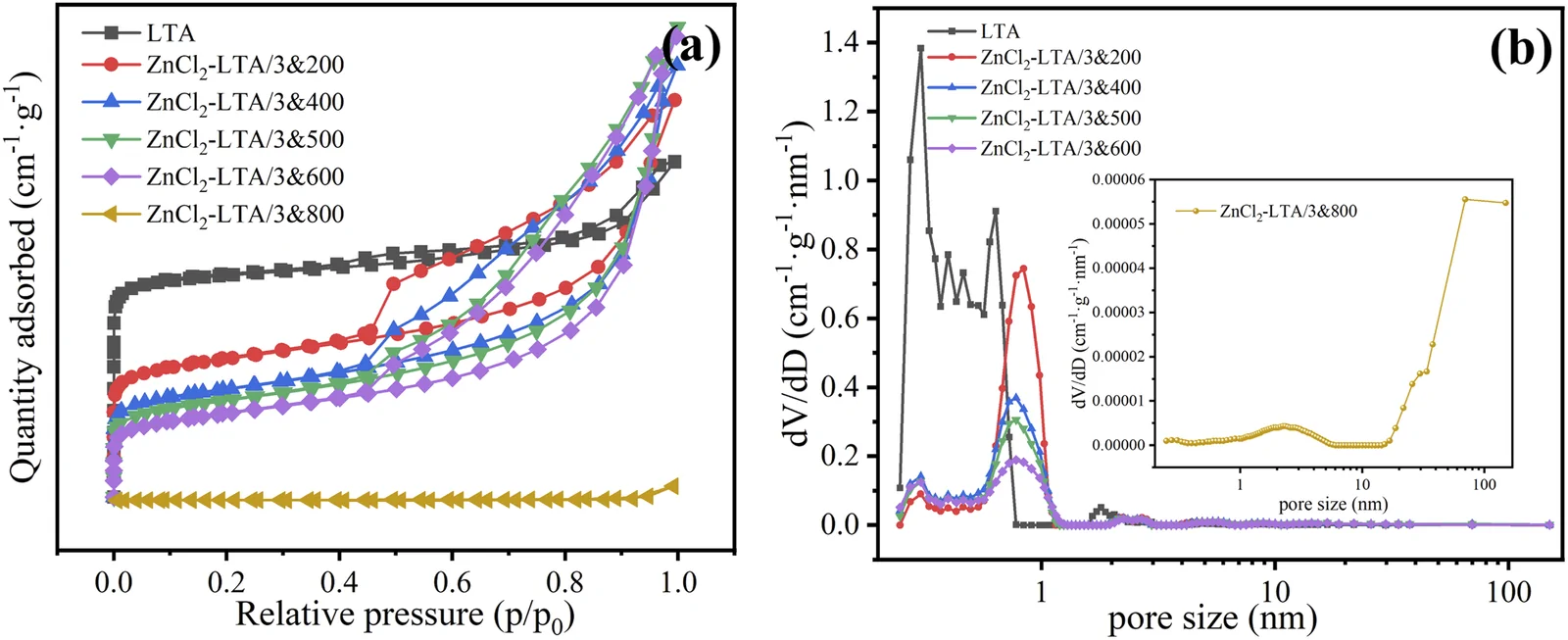
(a) N_2_ adsorption isotherms and (b) pore size distributions of the ZnCl_2_-LTA/3&*T* catalysts.

**Table 2:** Textural properties of ZnCl_2_-LTA/3&*T* catalysts

Samples	*S*_BET_ a (m^2^ g^−1^)	Pore volume (cm^3^ g^−1^)	Average pore size^b^ (nm)
LTA	449.61	0.26	1.17
ZnCl_2_-LTA/3&200	263.47	0.31	2.33
ZnCl_2_-LTA/3&400	204.59	0.33	3.22
ZnCl_2_-LTA/3&500	181.75	0.36	3.93
ZnCl_2_-LTA/3&600	155.60	0.35	4.55
ZnCl_2_-LTA/3&800	1.02	0.01	21.71

a Calculated by the BET method.

b Determined by the BJH model from the desorption branch.

The TGA curve for ZnX-LTA/3&400 is illustrated in Fig. S2. All samples show a weight loss of about 1–2% before reaching 100 °C, that mainly ascribed to the desorption of the deionized water. It should be noted that in LTA-type zeolites, the desorption of framework-associated water and hydrated cations can extend well beyond 200 °C, with significant mass loss events reported up to 300 °C.^32^ Between 100 °C and 200 °C, the weight loss becomes more pronounced, which may be attributed to the continued desorption of such tightly bound water species rather than framework decomposition.^33^ In particular, in the critical reaction temperature window for the PET glycolysis (150–200 °C), the catalyst prepared with the zinc precursor of ZnCl_2_ exhibits the smallest weight loss, that shows a better thermal stability under the operating condition, which is conducive to maintaining catalytic activity.

### 3.2. The effect of catalysts on glycolysis of PET

In order to evaluate systematically the catalytic performance of the Zn-modified 5A zeolite in the PET glycolysis, the reactions are performed under the same conditions (195 °C, 6 h, 10 g PET, 1 g catalyst). As it is shown in Fig. 4a, using ZnCl_2_ as the precursor with calcination at 400 °C, the PET conversion increased with ion exchange cycles until it gives a plateau after three cycles. The first two cycles give a suboptimal conversion efficiency (27.68% and 42.23% respectively), which means that the loading of Zn^2+^ in the zeolite framework is inadequate. When reach third exchange cycle, the conversion soared to 86.32% with 73.31% BHET yield. Afterward, in the other cycles (up to five) is not obtained any improvement, which means that the Zn^2+^ loading are saturated. Fig. 4b compares the catalytic performance of different zinc precursors under the same exchange cycles and calcination temperature. The catalyst with ZnCl_2_ as the zinc precursor exhibited the optimal catalytic activity, with 86.32% PET conversion and 73.31% BHET yield. This is superior than those catalyst with Zn(NO_3_)_2_ (29.14% of conversion) and Zn(CH_3_COO)_2_ (49.93% of conversion) as zinc precursor. This difference can be attributed to the influence of different anions on the solubility and dissociation behavior of zinc salts, which governs the availability of free Zn^2+^ ions in solution for ion exchange.^34^ The aqueous solubilities of the four zinc precursors at 25 °C follow the order ZnCl_2_ (432 g per 100 g) > Zn(NO_3_)_2_·6H_2_O (184 g per 100 g) > ZnSO_4_ (54.4 g per 100 g) > Zn(CH_3_COO)_2_·2H_2_O (43 g per 100 g). XRD and FTIR data (Fig. 1b and 2b) indicate that ZnCl_2_, owing to its higher solubility and stronger dissociation in the ion exchange medium, can more effectively release Zn^2+^ ions, which are then incorporated into the zeolite framework to form stable Lewis acid active centers.^35^Fig. 4c is showed the influence of calcination temperature in the catalytic performance. It can be seen that the calcination is not complete in 200 °C, the conversion was only 45.2%. As the calcination temperature increased to 400 °C and 500 °C, the conversion reached 86.32% and 87.58%, showing the best performance. However, when the calcination temperature is higher than 600 °C, the catalytic activity sharply decreased, even reaching only 20.21% conversion at 800 °C. Combining SEM, BET and XRD analyses, it can be confirmed that high temperatures caused serious sintering of the catalyst particles (Fig. S1), a sharp drop of the specific surface area from 204.59 to 1.02 m^2^ g^−1^ (Fig. 3a and Table 1), and the collapse of the microporous framework (Fig. 1c). These structural changes indicate that the Zn^2+^ active sites, which were originally distributed within the zeolite micropores and on the internal surface, become inaccessible due to pore collapse and particle agglomeration (the burial of active sites), thereby losing the catalytic functionality.

**Fig. 4:**
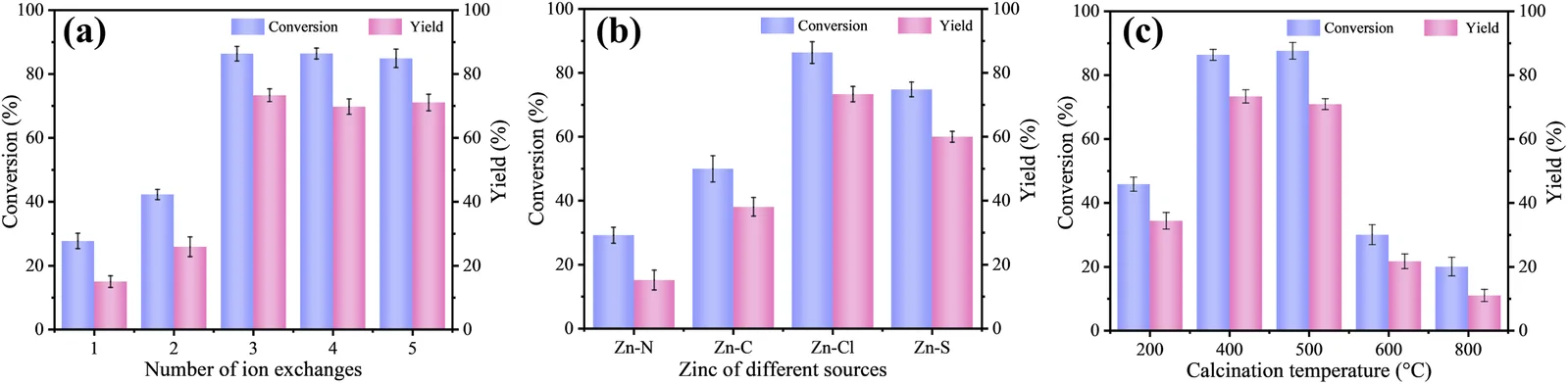
The glycolysis reaction on catalysts prepared with (a) number of ion exchanges; (b) zinc of different sources; (c) calcination temperature.

In order to improve glycolysis efficiency of PET, the effect of the process parameters (including reaction temperature, catalyst dosage, EG to PET mass ratio and reaction time) on the catalytic performance were also studied. As shown in Fig. 5a, the reaction temperature has important impact on the catalytic activity. Below 190 °C, the PET conversion was lower than 40%; increasing to 190 °C, 75.61% conversion was achieved with 62.38% BHET yield. When the temperature reached 195 °C, PET conversion further increased to 86.32% with 73.13% BHET yield. Those indicate that elevated temperatures enhance the solvation and activation of EG, effectively facilitating the nucleophilic attack on ester bonds.^36^ The reaction temperature was capped at 195 °C to prevent EG (bp 197.3 °C) evaporation, as EG functions as both reactant and solvent and its loss would alter the reactant ratio and pose safety concerns. Increasing the catalyst dosage from 0.5 g to 0.75 g (Fig. 5b), the PET conversion improved from 27.08% to 87.33% and the BHET yield increased from 16.59% to 74.23%. However, increasing the catalyst dosage higher than 0.75 g, the conversion decreased, probably because of particles aggregate, thereby produces mass transfer limitation and blockages of the active sites. The effect of the EG/PET mass ratio (Fig. 5c) shows that as the ratio increase from 2 : 1 to 4 : 1, the PET conversion is progressing enhanced; increase the ratio to 6 : 1, the conversion has no more change. This indicates that when EG/PET = 4, EG is in an adequate excess. The appropriate EG concentration promotes the PET chain swelling and nucleophilic attack.^37,38^ Finally, the influence of reaction time was examined (Fig. 5d), it shows that the PET conversion progressively increased between 4 and 6 h and reached a maximum conversion of 87.33% at 6 h. It means that the reaction was basically completed within 6 hours. When the reaction time was extended to 7 h and 8 h, the yield slightly decreased, probably because of a secondary reaction that involved in the lost of the BHET under high-temperature for a long time.

**Fig. 5:**
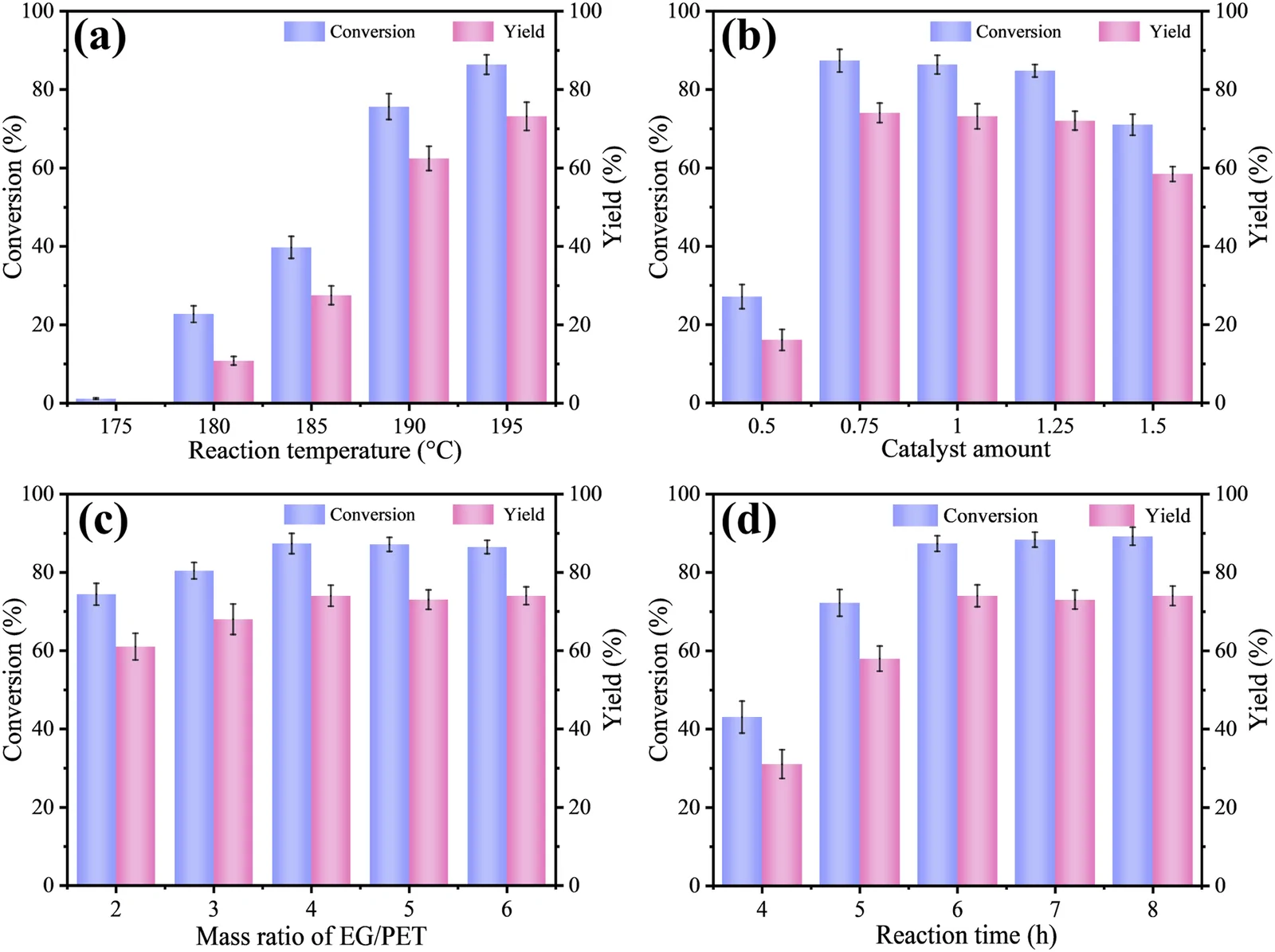
Effect of reaction conditions on PET glycolysis. (a) Reaction temperature (catalyst: 10 wt%, EG/PET = 4, 6 h); (b) catalyst amount (EG/PET = 4, 195 °C, 6 h); (c) mass ratio of EG/PET (catalyst: 7.5 wt%, 195 °C, 6 h); (d) reaction time (catalyst: 7.5 wt%, EG/PET = 4, 195 °C).

### 3.3. Recycle study of ZnCl_2_-LTA/3&400 catalyst for glycolysis

The recycling experiments were carried out on the best ZnCl_2_-LTA/3&400 catalyst with same reaction conditions (195 °C, 6 h, 0.75 g catalyst, EG/PET mass ratio = 4). After each cycle, the catalyst was separated by filtration, washed with deionized water and oven dried at 100 °C for 12 h, subsequently used in the next reactions. As shown in Fig. 6, the catalyst shows very exceptional stability in four cycles: the PET conversion remained above 80% and BHET yield stayed above 70%, and without significant decline. By the fifth cycle, the conversion slightly dropped to 75.68% with 62.48% BHET yield. Inductively coupled plasma mass spectrometry (ICP-MS) analysis was performed on both the fresh and the reused ZnCl_2_-LTA/3&400 catalyst. As summarized in Table S3, the Zn content in the fresh catalyst was determined to be 2.47 wt%. After five recycling cycles, the Zn content decreased only slightly to 2.29 wt%, corresponding to a Zn retention of 92.71%. These results confirm that the ion-exchange strategy successfully prevents the loss of active components during the glycolysis reaction, thereby ensuring the long-term stability of the catalyst.

**Fig. 6:**
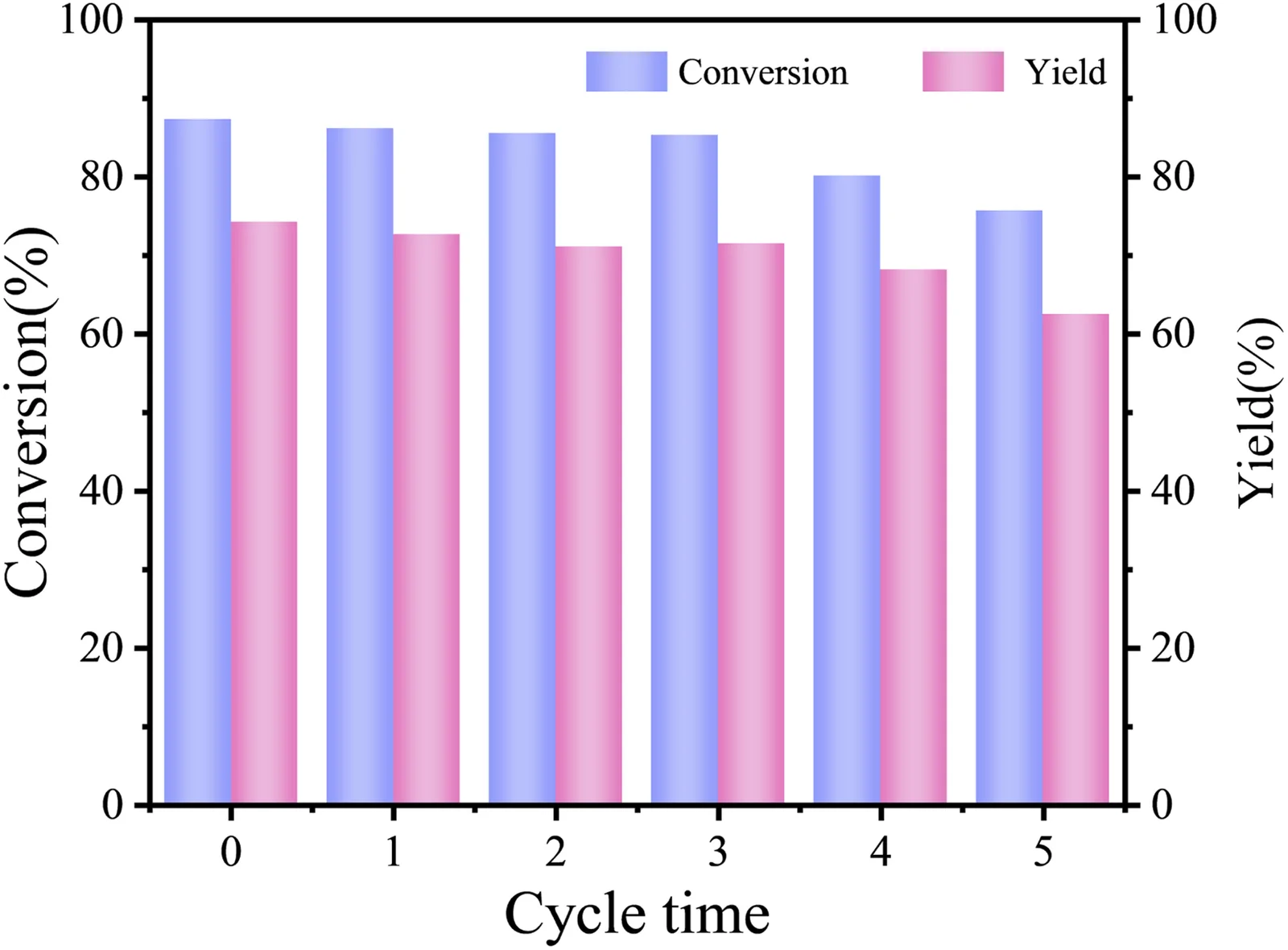
Catalyst recycling in PET glycolysis applying ZnCl_2_-LTA/3&400.

### 3.4. Kinetic study of glycolysis

A comprehensive kinetic analysis was performed to clarify intrinsic mechanism of the PET glycolysis reaction. This study adopted the shrinking core model^39–41^ for kinetic characterization due to the continuous consumption of PET particles and the gradual decrease in their geometric dimensions during the reaction. This model is based on four fundamental assumptions: (1) PET particles can be regarded as solid spheres; (2) glycolysis reaction occurs solely at the external surface of PET particles while retaining their spherical structure; (3) EG consumption is directly proportional to the surface area of unreacted PET particles; and (4) the produced BHET promptly dissolves in the EG medium without impeding the reaction progress.

With these assumptions, the reaction kinetics can be described by the following equation (see SI for the full derivation):

3 *Kt* = 1 − (1 − *X*)^2/3^

where *X* is the PET conversion, *t* is the reaction time, and *K* is the apparent rate constant, which is related to the diffusion coefficient (*D*) of EG at the reaction interface.

Fig. 7a illustrates excellent linear correlation in the 448.15–468.15 K, and the values of correlation coefficient (*R*^2^) are always greater than 0.98. This means close agreement of the experimental data with the shrinking core model. There is a strong linear relation between ln *K* and 1/*T* (Fig. 7b). The calculated apparent activation energy (*E*_a_) is 184.41 kJ mol^−1^, and the reported values of the similar systems (Na_2_CO_3_ catalytic system:^42^ 185.01 kJ mol^−1^; CoFe_2_O_4_ spinel system:^43^ 188.01 kJ mol^−1^) are very close to this value, which verify the reliability of shrinking core model. The higher activation energy indicates that the reaction needs to overcome a larger activation energy barrier and is a typical endothermic reaction. The SEM micrographs (Fig. 7c and d) show that original PET particles have well defined geometric morphology, although the residues of post-reaction have reduced in size, their overall shape remains intact and no obvious fragmentation or disintegration has occurred. This further confirms that the reaction begins on the surface of the PET particles and gradually progresses towards the inner core, which is in line with the shrinking core mechanism.

**Fig. 7:**
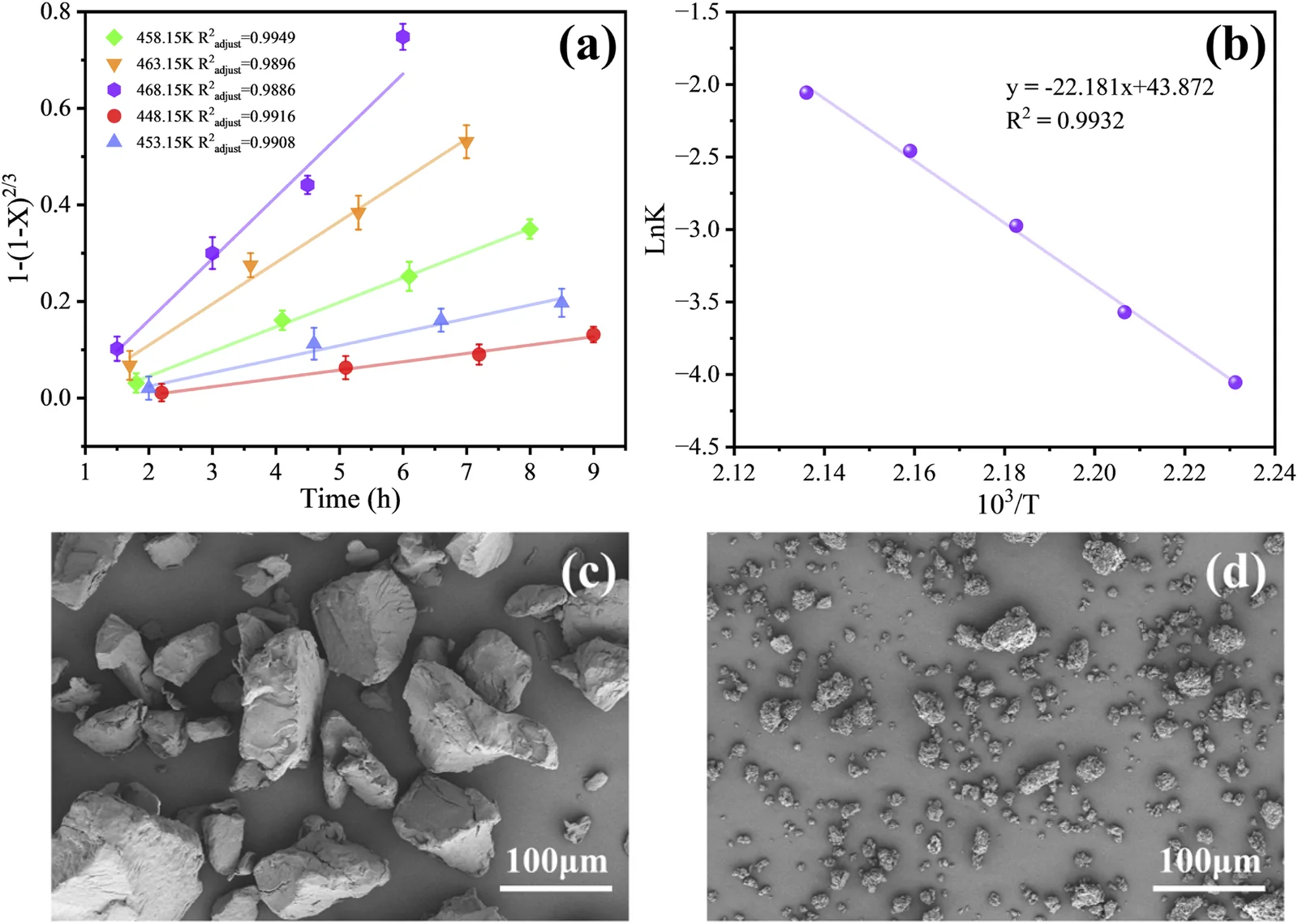
Kinetic analysis and morphological evolution of PET glycolysis: (a) shrinking core model fitting curves at different temperatures; (b) Arrhenius plot; SEM image of (c) fresh PET particles and (d) residual PET particles after reaction.

### 3.5. Glycolysis of PET from different sources

To assess the practical applicability of the developed catalyst for the waste PET recycling, glycolysis experiments of four typical PET materials (pure PET pellets, PET tray, beverage bottle, and PET film) with EG were carried out. All the reactions were performed in the optimized condition (195 °C, 6 h, 0.75 g catalyst, EG/PET mass ratio = 4). As shown in Fig. 8, the differences of the conversion and the BHET yield among PET samples from various sources. The PET film has best performance with the complete conversion (100%) and BHET yield of 81.93%. Both PET tray and beverage bottle also have more than 90% of the conversion. The results are better than those reported in most previous studies on the glycolysis of waste PET (Fig. S3). Collectively, it shows that the post-consumer PET generally have higher glycolytic reactivity than pure PET. The reaction kinetics for each sample was also analyzed with the shrinking core model (Fig. S4a). The data obtained have the good linear correlations in all the specimens. The calculated apparent rate constants (*K* values) are presented in Fig. S4b. It can be seen that the PET tray and film have best reaction rate of 0.1928 h^−1^ and 0.1908 h^−1^ respectively, followed by PET beverage bottles, while pure PET has the slowest reaction rate at 0.1502 h^−1^. Those results were consistent with the actual trend of reaction conversion. The kinetic differences mainly attributed to the physical structure and crystallinity of the material. PET films usually have low crystallinity and large specific surface area, which is conducive to the diffusion of EG molecules and the occurrence of catalytic reactions;^44^ although PET trays and beverage bottles are produced through injection molding, their surfaces have numerous micropores and stress cracks, which also enhance the mass transfer efficiency;^45,46^ on the contrary, pure PET particles are highly crystalline materials with tightly arranged molecular chains, which restrict the diffusion of EG and result in low reaction activity.^47^

**Fig. 8:**
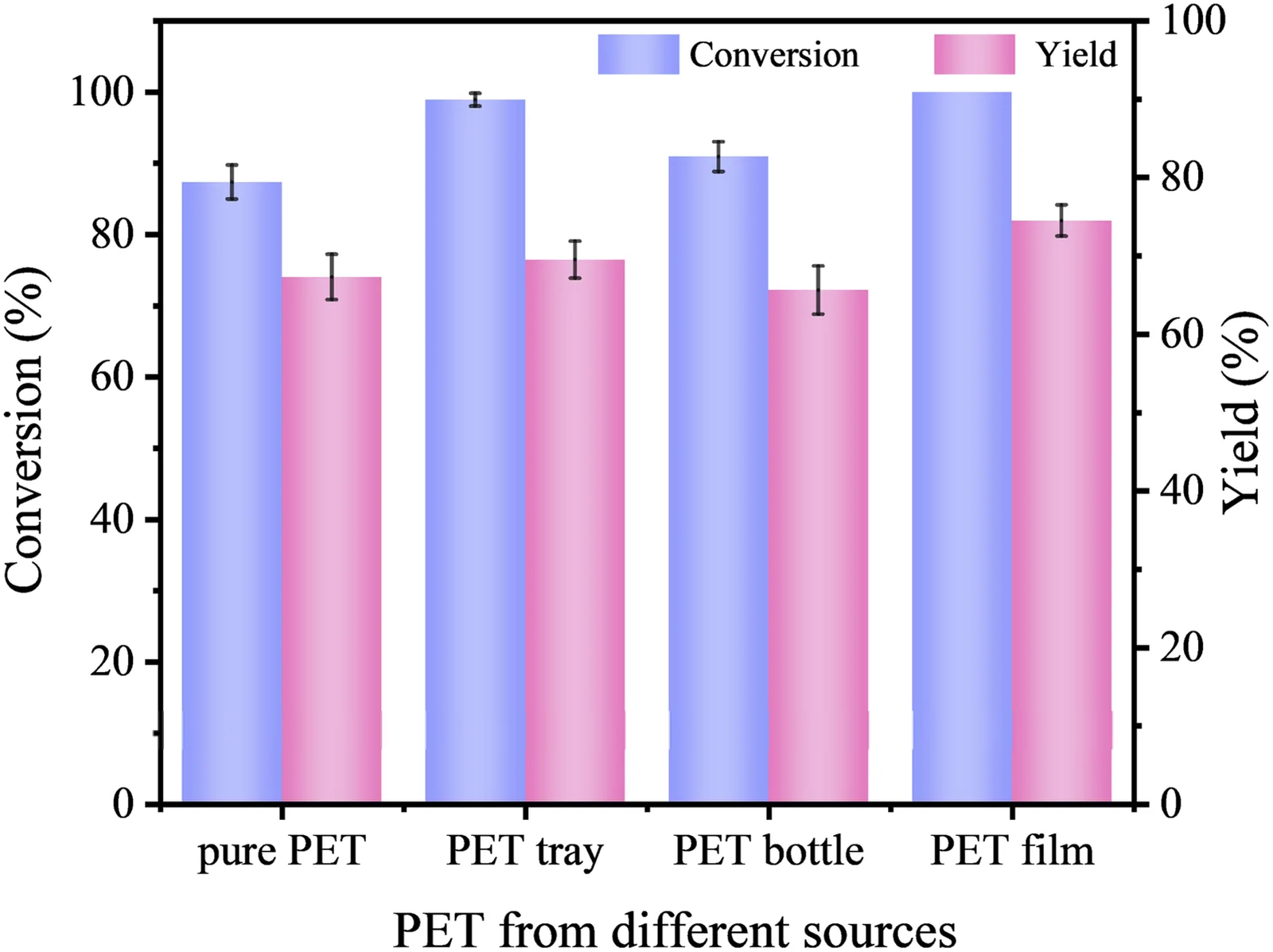
PET conversion and BHET yield from different PET sources.

### 3.6. PET glycolysis mechanism

Zn^2+^ were introduced into the structure of 5A zeolite (LTA) *via* ion exchange, displacing native Ca^2+^ ions to form Zn^2+^ active centers. The introduced Zn^2+^ coordinate with the framework oxygen atoms, contributing to the thermal stability of the catalyst at the high temperature. Based on the structural characterization of the catalyst and the literature reports,^48^ in the glycolysis reaction, the ester groups in the PET long chains are close to the catalyst surface. The carbonyl oxygen (C=O) of these ester groups is rich in lone pairs of electrons, which form coordination bonds with Zn^2+^ (Lewis acid) as shown in Fig. 9. This coordination significantly enhances the polarity of the C=O bond, further increasing the positive charge of carbonyl carbon atom, while weakening the adjacent acyl–oxygen (C–O) bond, thereby activating the ester group. Afterwards, the nucleophilic hydroxyl oxygen from the EG molecules attack the activated carbonyl carbon. At the same time, the O–H bond of EG undergoes polarization and even partial dissociation under the Zn^2+^ synergic effects, releasing protons (H^+^), which promotes the cleavage of the C–O bond. As new C–O bonds are formed between EG and the carbonyl carbon, the C–O bonds in the original ester group cleaves, causing the PET long chain to depolymerize into short chain fragments. These short chain fragments continue to depolymerize to oligomers, until they finally depolymerize to BHET monomers.

**Fig. 9:**
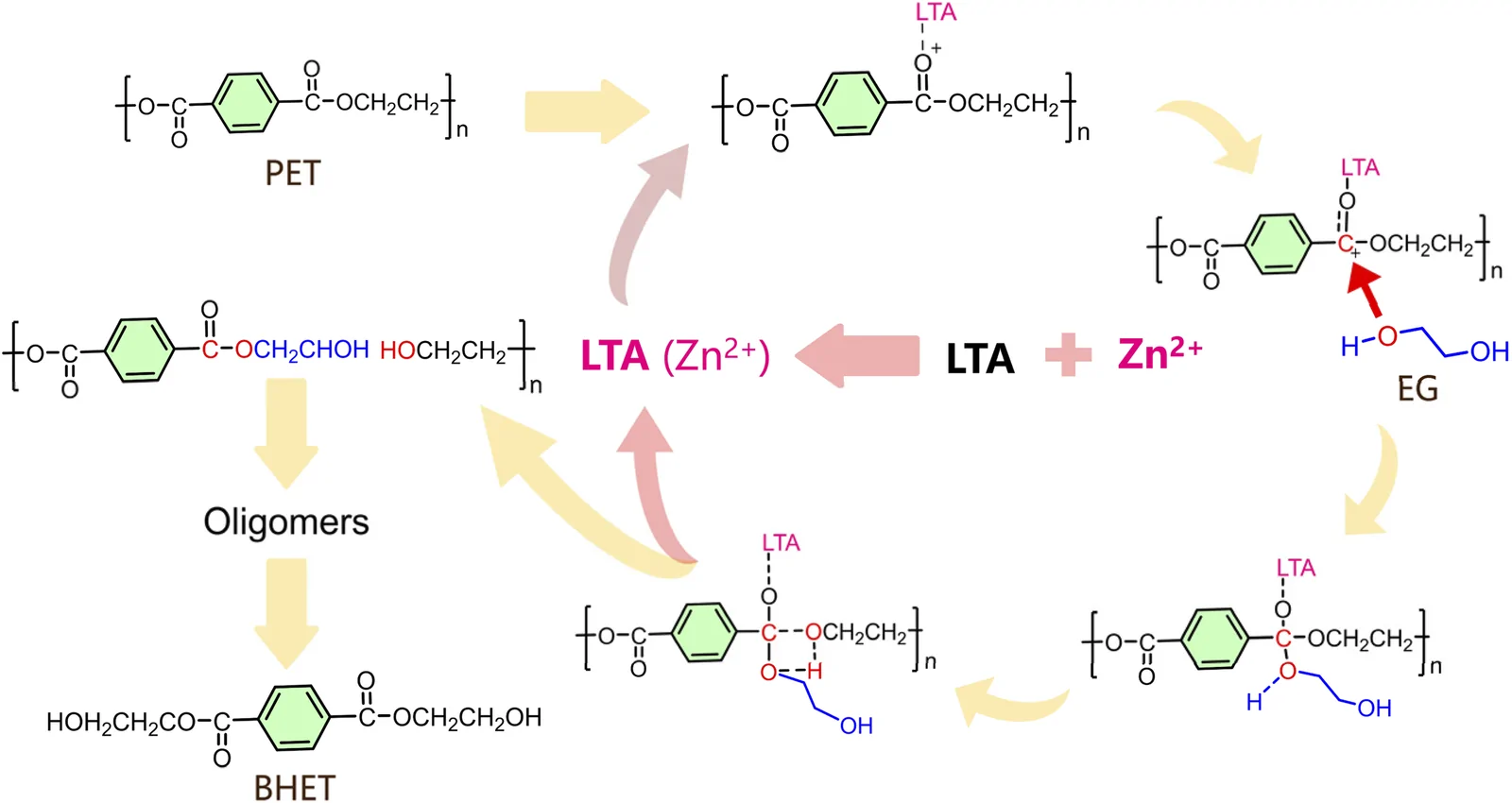
Proposed PET glycolysis mechanism over ZnCl_2_-LTA/3&400 catalyst.

## 4. Conclusion

In this study, a series of Zn-modified 5A zeolite catalysts (ZnX-LTA/*N*&*T*) were prepared *via* an ion-exchange method. The optimal catalyst, designated ZnCl_2_-LTA/3&400, was prepared by using ZnCl_2_ as the zinc precursor, being calcined at 400 °C and undergoing three exchange cycles. It effectively overcomes the defects associated with conventional catalyst preparation methods, including high costs and susceptibility to active site deactivation. The PET conversion reached 87.33% with 74.23% BHET yield, and the activity showed no obvious decline after 5 recycling cycles. The kinetic analysis showed that the glycolysis reaction adhered to the shrinking core model with apparent activation energy of 184.41 kJ mol^−1^. SEM observations confirmed the model mechanisms that gradual depolymerization from the surface of the particles inward. In this catalyst, it was also showed outstanding universality for real waste PET (films, trays and beverage bottles). Among them, the PET films achieved complete PET conversion (100%) with 81.93% of BHET yield. These results show its potential for the industrial scale of the chemical recycling, and has an efficient and stable path for high value of the use of the PET waste.

## Conflicts of interest

There are no conflicts to declare.

## Supplementary Material

RA-OLF-D6RA08028F-s001

## Data Availability

Data will be made available on request. Supplementary information (SI) is available. See DOI: https://doi.org/10.1039/d6ra08028f.
