# Supplementary material for: Highly efficient glycolysis of polyethylene terephthalate catalyzed by a Zn-modified 5A zeolite prepared through ion exchange
Source: RSC Adv. 2026 Nov 4. Online ahead of print. doi: 10.1039/d6ra08028f (PMC13733665; doi:10.1039/d6ra08028f)
Supplement: Supplementary file 1 [file RA-OLF-D6RA08028F-s001.pdf]

Supplementary Information (SI) for RSC Advances.
This journal is © The Royal Society of Chemistry

Supporting Information

Highly efficient glycolysis of polyethylene terephthalate catalyzed by a Zn-modified 5A zeolite prepared through ion exchange

Haibo Wang[a,b,c,#], Xiuqi Wang[b,c,#], Zixin Lin [a,*], Haoyu Yao [b,c,*]

[a] Key Laboratory of Marine Chemistry Theory and Technology (Ministry of Education), College of Chemistry and Chemical Engineering, Ocean University of China, Qingdao, China

[b] Qingdao Institute of Bioenergy and Bioprocess Technology, Chinese Academy of Sciences, Qingdao, China

[c] Shandong Energy Institute, Qingdao, China

[#] These authors contributed equally to this work

[*] Corresponding Author: Zixin Lin, email: linzixin@ouc.edu.cn

Haoyu Yao, email: yaohy@qibebt.ac.cn

Table S1. Preliminary cost analysis of the $ZnCl_2$-LTA/3&400 catalyst

Cost Item	Quantity per kg catalyst	Unit Price ($/unit)	Cost ($)
5A Zeolite (LTA) support	1.00 kg	~3.50/kg (commercial)	$3.50
$ZnCl_2$ (precursor)	0.05 kg (50 g)	~2.50/kg (reagent grade)	$0.13
Energy (calcination @ 400 °C, 6 h)	—	estimated ~$0.50/kg	$0.50
Total estimated preparation cost			$4.13

All prices are based on 2024-2025 commercial market rates

Table S2. Cost comparison of zinc-based PET glycolysis catalysts

Catalyst	Support	Preparation Method	Cost ($/kg)	Ref
**$ZnCl_2$-LTA/3&400**	LTA zeolite	Ion exchange + calcination (400°C)	~4	This work
ZnO/SBA-15	SBA-15 (molecular sieve)	Template-assisted synthesis	~20 (estimated)	1
Zn/MCM-41	MCM-41 (molecular sieve)	Impregnation	~18 (estimated)	2

$ZnCl_2$/ZIF-8	ZIF-8 (MOF)	Solvothermal + calcination	~60 (estimated)	3

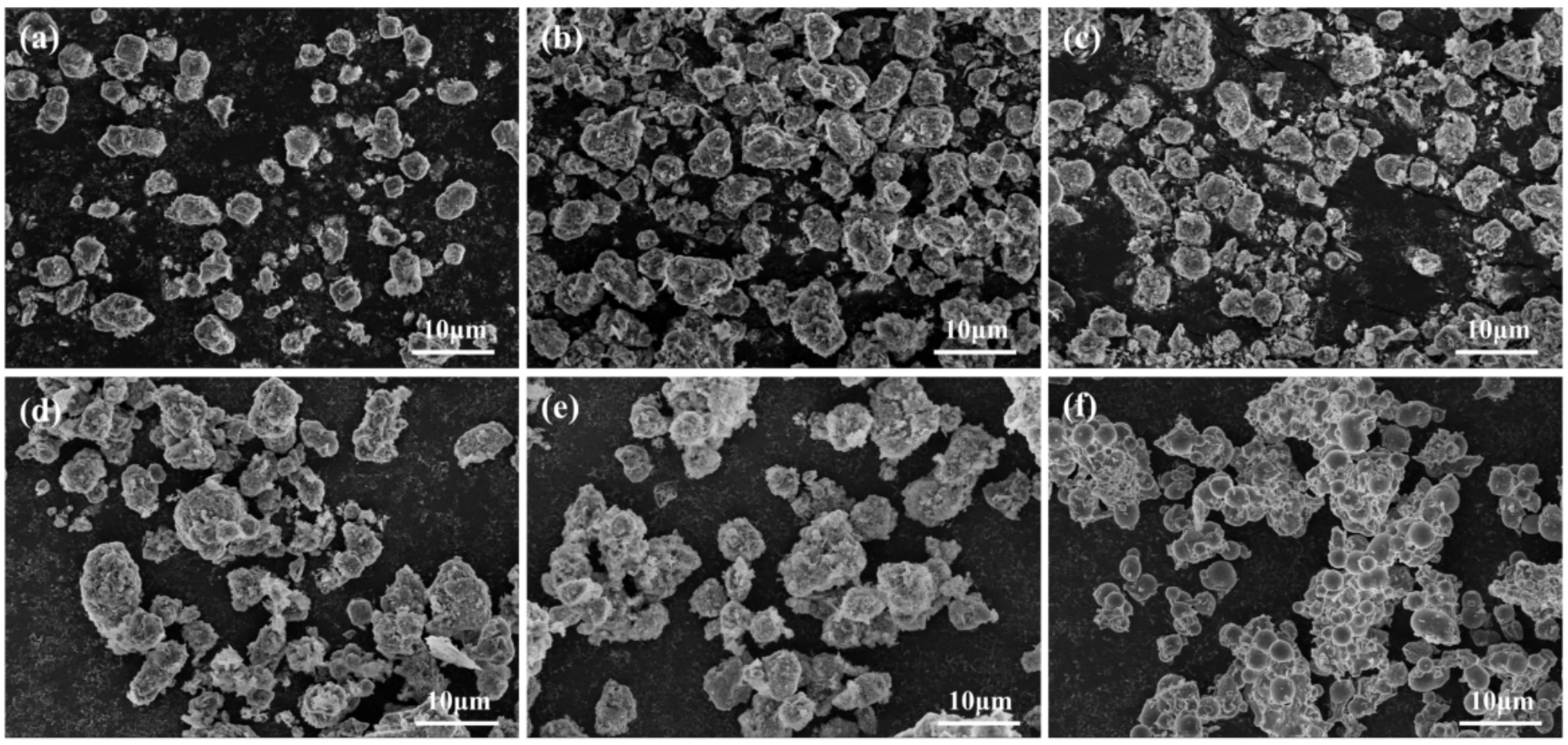

Figure S1. SEM images of (a) the LTA and $ZnCl_2$-LTA/3 with different calcination temperatures: (b) $ZnCl_2$-LTA/3&200; (c) $ZnCl_2$-LTA/3&400; (d) $ZnCl_2$-LTA/3&500; (e) $ZnCl_2$-LTA/3&600; and (f) $ZnCl_2$-LTA/3&800.

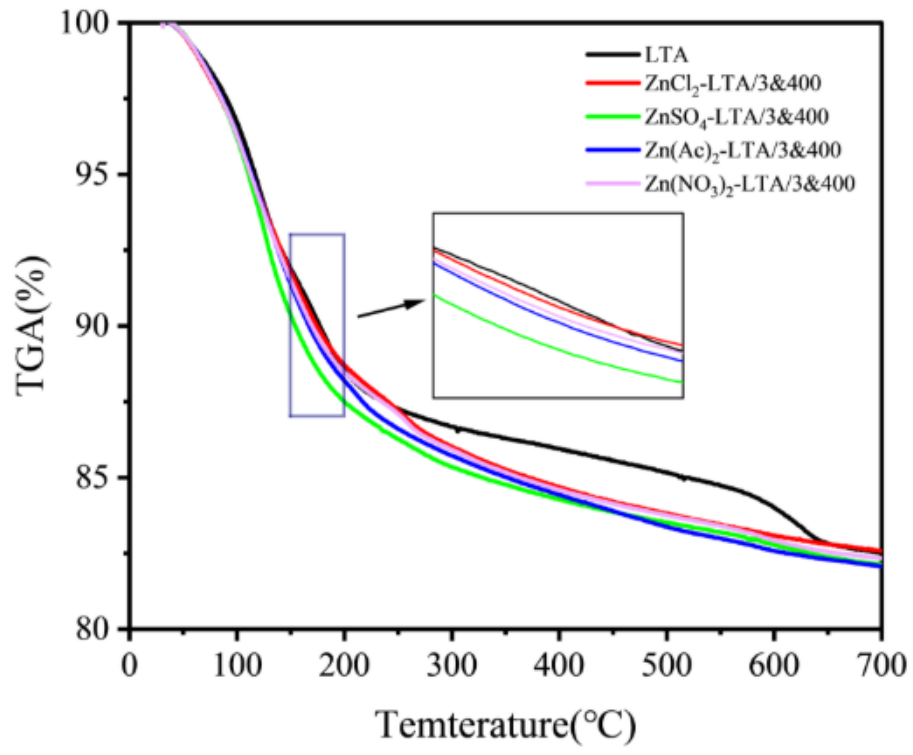

Figure S2. TGA curves of 5A catalysts modified with different zinc sources.

The metal content of the fresh and spent catalysts was determined using inductively coupled plasma mass spectrometry (ICP-MS, Agilent 7850). Approximately 50 mg of the catalyst sample was digested in HNO_3 in a microwave digestion system at 180 °C for 20 min. The resulting solutions were diluted to 50 mL with deionized water prior to analysis. Additionally, the liquid product obtained after the glycolysis reaction was filtered through a 0.22 μm membrane and directly analyzed to detect any leached metal species. The results are summarized in Table S3.

Table S3 ICP-MS analysis of Zn and Al content in fresh and reused $ZnCl_2$-LTA/3&400 catalysts

Sample	Zn (wt%)	Al (wt%)	Zn retention (%)
Fresh	2.47	5.82	100.00
After 5 cycles	2.29	5.79	92.71

S1. Kinetic model derivation for PET glycolysis

To elucidate the reaction mechanism of PET glycolysis catalyzed by $ZnCl_2$-LTA/3&400, a kinetic analysis based on the shrinking core model was conducted. The shrinking core model assumes that the reaction occurs at the surface of unreacted PET particles, and the product (BHET) dissolves immediately into the EG medium without forming a solid layer on the particle surface. The following assumptions were made:

1. PET particles can be regarded as solid spheres;
2. Glycolysis reaction occurs solely at the external surface of PET particles while retaining their spherical structure;
3. EG consumption is directly proportional to the surface area of unreacted PET particles;
4. Produced BHET promptly dissolves in the EG medium without impeding the reaction progress.

S1.1. Shrinking core model equation

Based on these assumptions, the kinetic equation for the reaction can be derived. Let r_x represent the radius of unreacted PET particles and r_0 denote the initial radius. The rate of mass change for unreacted PET is then expressed as:

$$\frac{\square\square_{PET}}{\square\square} = -\square\frac{\square}{\square\square}\left(\frac{4}{3}\square\square_{\square}^{3}\right) \tag{1}$$

Where $\square$ denotes the density of PET. Given that the EG consumption rate is proportional to the surface area of unreacted PET particles, expressed as $S_{PET} = 4\square\square_{\square}^{2}$, the reaction kinetics can be formulated as $\frac{\square\square_{EG}}{\square\square} = \square\square C_{EG} S_{PET}$, By integrating this equation with the mass conservation principle $\frac{\square\square_{PET}}{\square\square} = \square\frac{\square\square_{PET}}{\square\square}$, the following relationship is derived:

$$-\square\frac{\square}{\square\square}\left(\frac{4}{3}\square\square_{\square}^{3}\right) = \square\square C_{EG}4\square\square_{\square}^{2} \quad (2)$$

Upon completion, the following results were obtained:

$$\frac{\square\square_{\square}}{\square\square} = -\frac{\square\square C_{EG}}{\square\square_{\square}} \quad (3)$$

By integrating and introducing the PET conversion rate $X = 1 - \frac{4/3\square\square_{\square}^{3}}{4/3\square\square_{\circ}^{3}} = 1 - \left(\frac{\square_{\square}}{\square_{\circ}}\right)^{3}$, the simplified form of the shrinking-core model can be ultimately derived:

$$K\square = 1 - (1 - X)^{2/3} \quad (4)$$

The apparent rate constant in the equation, $K = \frac{2\square D C_{EG}}{\square\square_{\circ}^{2}}$ is characterized by the diffusion coefficient (D) of EG at the reaction interface.

The complete dataset, including reaction time, PET conversion, and the calculated values of $1 - (1 - X)^{2/3}$, is provided in Table S4 below.

S1.2. Activation energy determination

The temperature dependence of the rate constant K follows the Arrhenius equation:

$$K = A\exp\square\left(-\frac{Ea}{RT}\right) \quad (5)$$

Where A is the pre-exponential factor, Ea is the apparent activation energy, R is the gas constant (8.314 $J\cdot mol^{-1}\cdot K^{-1}$), and T is the absolute temperature in Kelvin.

By plotting lnK versus 1/T, the activation energy (Ea) can be determined from the slope of the linear fit.

Table S4. Detailed kinetic data for PET glycolysis at different temperatures.

Temperature (°C)	Time (h)	$1-(1-X)^{2/3}$ mean
175	2.2	0.0106
	5.1	0.0625
	7.2	0.0897
	9.0	0.1311
180	2.0	0.0201
	4.6	0.1121
	6.6	0.1609
	8.5	0.1968
185	1.8	0.0307
	4.1	0.1608
	6.1	0.2515
	8.0	0.3494
190	1.7	0.0673
	3.6	0.2747
	5.3	0.3837
	7.0	0.5306
195	1.5	0.1016
	3	0.2998
	4.5	0.4411
	6.0	0.6929

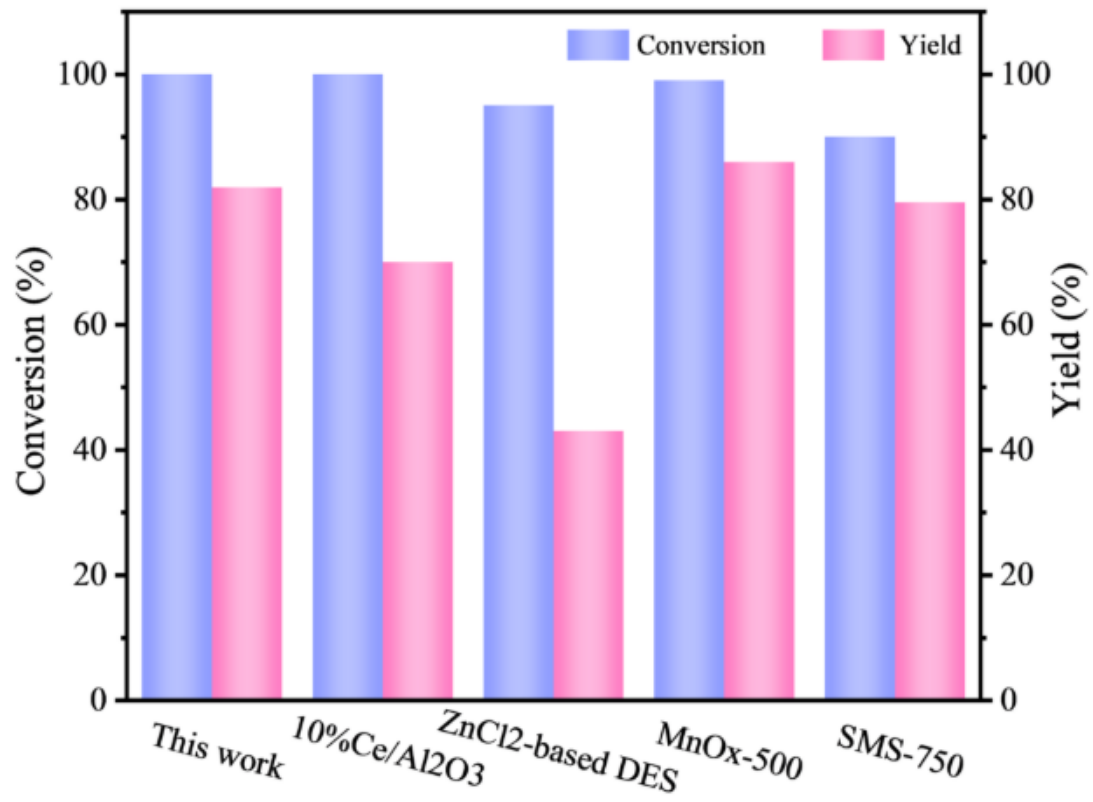

Figure S3. Catalytic performance comparison of the $ZnCl_2$-LTA/3&400 catalyst (This work) with other recently reported systems for PET glycolysis. Literature data are derived from: 10%Ce/Al_2O_3[4], $ZnCl_2$-based DES[5], MnO_x-500[6], and SMS-750[7].

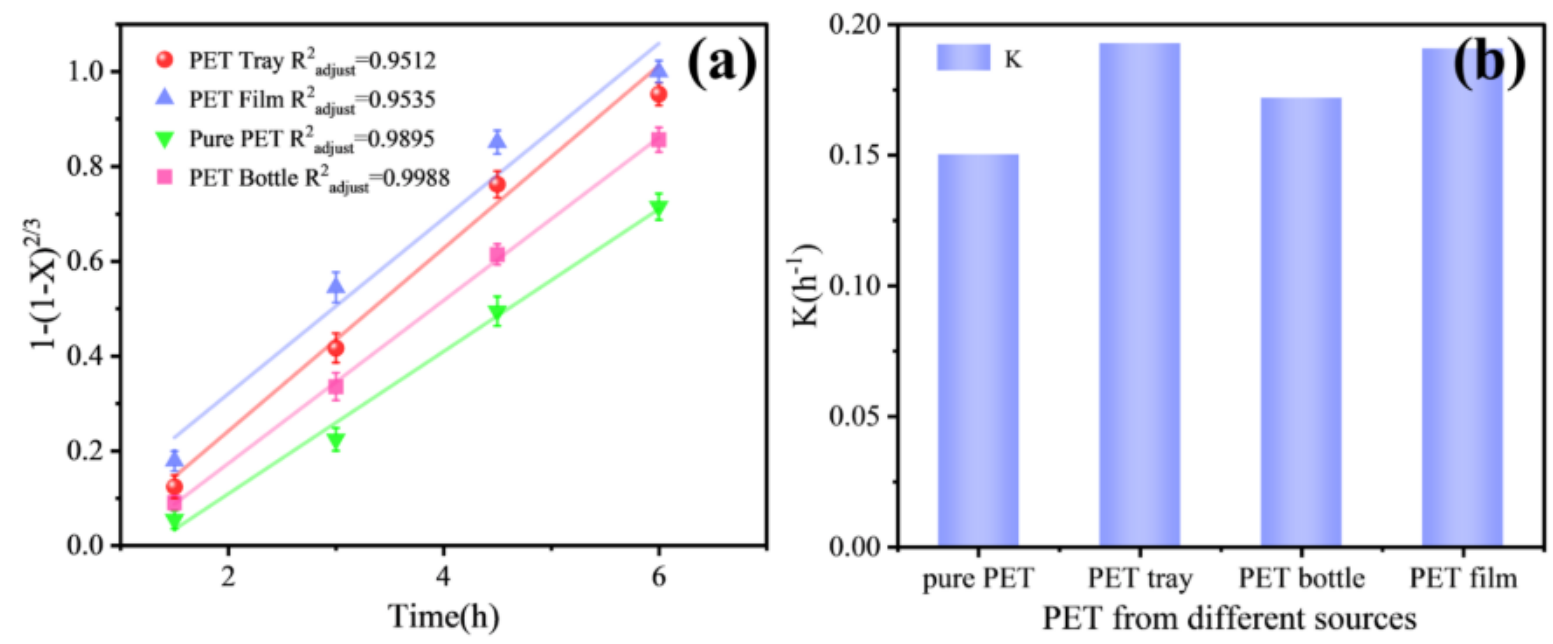

Figure S4. Kinetic analysis of PET glycolysis from different sources: (a) Shrinking core model fitting curves; (b) Comparison of apparent rate constants (K).

References

(1) Yao, H.; Liu, L.; Yan, D.; Zhou, Q.; Xin, J.; Lu, X.; Zhang, S. Colorless BHET Obtained from PET by Modified Mesoporous Catalyst ZnO/SBA-15. *Chemical Engineering Science* **2022**, *248*, 117109.

(2) Dai, C.; Liu, Y.; Wang, Z.; Yu, G. Efficient Glycolysis of Waste Polyethylene Terephthalate Textiles over Zn-MCM-41 Catalysts. *Catalysis Today* **2024**, *440*, 114827.

(3) Wang, R.; Wang, T.; Yu, G.; Chen, X. A new class of catalysts for the glycolysis of PET: Deep eutectic solvent@ZIF-8 composite. *Polymer Degradation and Stability* **2021**, *183*, 109463.

(4) Pham, D. D.; Cao, A. N. T.; Senthil Kumar, P.; Nguyen, T. B.; Tran Nguyen, H.; Phuong, P. T. T.; Nguyen, D. L. T.; Nabgan, W.; Trinh, T. H.; Vo, D.-V. N. Insight the Influence of the Catalyst Basicity on Glycolysis Behavior of Polyethylene Terephthalate (PET). *Chemical Engineering Science* **2023**, *282*, 119356.

(5) Li, Y.-C.; Lu, P.-Y.; Lin, Y.-C.; Chen, W.-C. Closed-Loop Recycling of Polyesters through Glycolysis Using Catalytic Deep Eutectic Solvents and the Influence of Glycol Types on Reaction Kinetics. *Polymer Degradation and Stability* **2026**, *245*, 111918.

(6) Swapna, B.; Singh, N.; Patowary, S.; Bharali, P.; Madras, G.; Sudarsanam, P. Efficient Glycolysis of Used PET Bottles into a High-Quality Valuable Monomer Using a Shape-Engineered MnOx Nanocatalyst. *Catalysis Science & Technology* **2024**, *14* (19), 5574–5587.

(7) Zhao, L.; Shen, G.; Wen, R.; Xu, T.; Jiang, S.; Wang, X.; Wang, H. Optimization of Operating Conditions for the Catalytic Alcoholysis of Waste PET for the Synthesis of

BHET by Sunflower Seed Husk Matrix Materials. *RSC Advances* **2025**, *15* (1), 207–215.
